# Characterisation of genes differentially expressed in macrophages by virulent and attenuated ***Mycobacterium tuberculosis*through RNA-Seq analysis**

**DOI:** 10.1038/s41598-019-40814-0

**Published:** 2019-03-11

**Authors:** Junghwan Lee, Sung-Gwon Lee, Kee K. Kim, Yun-Ji Lim, Ji-Ae Choi, Soo-Na Cho, Chungoo Park, Chang-Hwa Song

**Affiliations:** 1 Department of Microbiology, College of Medicine, Chungnam National University, Daejeon, 35015 South Korea; 2 Department of Medical Science, College of Medicine, Chungnam National University, Daejeon, 35015 South Korea; 3 Research Institute for Medical Sciences, College of Medicine, Chungnam National University, Daejeon, 35015 South Korea; 4 Department of Biochemistry, College of Natural Sciences, Chungnam National University, Daejeon, 34134 South Korea; 5 School of Biological Sciences and Technology, Chonnam National University, Gwangju, 61186 South Korea

## Abstract

Tuberculosis (TB) remains a global healthcare issue. Understanding the host-pathogen interactions in TB is vital to develop strategies and therapeutic tools for the control of *Mycobacterium tuberculosis* (Mtb). In this study, transcriptome analyses of macrophages infected with either the virulent Mtb strain H37Rv (Rv) or the avirulent Mtb strain H37Ra (Ra) were carried out and 750 differentially expressed genes (DEGs) were identified. As expected, the DEGs were mainly involved in the induction of innate immune responses against mycobacterial infections. Among the DEGs, *solute carrier family* 7 *member* 2 (*Slc*7*a*2) was more strongly expressed in Ra-infected macrophages. Induction of SLC7A2 was important for macrophages to control the intracellular survival of Mtb. Our results imply that SLC7A2 plays an important role in macrophages during Mtb infection. Our findings could prove useful for the development of new therapeutic strategies to control TB infection.

## Introduction

Tuberculosis (TB) caused by *Mycobacterium tuberculosis* (Mtb) is one of the main infectious diseases in the world^1^. TB is a major cause of death in many countries^2^. The development of TB appears to be related to the pathogenicity of Mtb in the host^3^.

It has been suggested that Mtb regulates cell death to enable it to survive in host cells^4^. The avirulent Mtb strain H37Ra (Ra) increases the synthesis of cyclooxygenase 2 (COX2), causing induction prostaglandin E_2_ (PGE_2_) in infected cells^5^. PGE_2_ is involved in protection of the mitochondrial inner membrane, repair of plasma membrane damage, and inhibition of lipoxin A_4_ (LXA_4_)^6^. Its effects counteract necrosis and trigger apoptosis in macrophages infected with Ra^7,8^. By contrast, the virulent Mtb strain H37Rv (Rv) increases production of LXA_4_, which prevents COX2 mRNA expression^6^. Rv reduces production of PGE_2_, leading to necrosis in infected macrophages^8,9^. Therefore, apoptosis is considered a host defence mechanism against Mtb infection. However, it remains unclear why immune responses to the virulent Mtb strain differ from those to the avirulent Mtb strain.

Rv strongly induces phosphorylation of ESX-1 secretion-associated proteinJ (EspJ), which is predicted to be a virulent factor of Mtb^10^. Rv also increases expression of microRNA-132, which prevents development of proinflammatory cytokines in human monocyte-derived dendritic cells^11^. It is evident that gene expression differs in macrophages infected with various mycobacterial strains. Nevertheless, it is still not fully clear which gene is important for host protection against Mtb infection. Therefore, comparative studies of virulent and avirulent strains of Mtb are essential to aid our understanding of the pathogenesis of TB.

RNA sequencing (RNA-Seq) is useful for measuring RNA expression, discovering small RNAs, and detecting new genes that respond to various stimuli^12,13^. In studies of pathogenic diseases, RNA-Seq has been used to reveal changes in gene expression for infectious bacteria, viruses, and fungi^14–16^. RNA-Seq analyses of TB have focused mainly on the transcriptome of the pathogenic Mtb, including profiles of particular environments and non-coding RNA, and have not focused on host cells^17–19^. Moreover, comparative gene expression analyses between Rv and Ra have been limited to variations in the bacterial gene sequences or expression^20–22^. To understand the interactions of Mtb and the host immune cells, transcriptome differences in macrophages infected with virulent or avirulent Mtb strains must be clarified.

RNA-Seq revealed that expression of *Slc7a2* appeared to be strongly suppressed in Rv-infected bone-marrow-derived macrophages (BMDMs), so the role of SLC7A2 in macrophages during Mtb infection was investigated.

## Results

### Genome-wide transcriptome analysis

To investigate global gene expression patterns and induction of the innate immune response in BMDMs infected with Rv or Ra, we performed genome-wide expression analysis using RNA-Seq. An average of 75.6 million raw sequencing reads (approximately 7.6 billion base pairs; average 2.7× genome coverage per sample) were generated from samples from three independent experiments (BMDMs without Mtb infection, with Rv infection, or with Ra infection), each with two biological replicates (Table 1 and Table S1). After trimming the raw sequence reads, in total 420 million (average 70 million) high-quality clean reads were mapped to the mouse reference genome, and between 63.2% and 86.1% were then uniquely mapped (Table 2). With a threshold of ≥1 fragment per kilobase of transcript per million mapped reads (FPKM), we detected 9,809 genes expressed in the control BMDMs (UN; unstimulated control); 9,492 for Ra infection; and 9,628 for Rv infection. To assess the reproducibility of our data, we calculated correlations across the biological replicates and found high correlations (Spearman’s correlation coefficient, mean ρ = 0.9707 ± 0.009; Table S1), implying that the results were highly reproducible.

**Table 1:** Raw reads statistics.

Sample	Replicate	Total read bases	Total reads	GC (%)	Q20 (%)	Q30 (%)
Control (UN)	1	7,616,860,662	75,414,462	54.75	96.54	94.16
	2	7,949,367,206	78,706,606	52.50	96.98	94.84
Infection (Ra)	1	7,046,020,580	69,762,580	61.00	95.72	92.77
	2	7,813,362,626	77,360,026	52.66	97.00	94.88
Infection (Rv)	1	7,180,698,222	71,096,022	56.54	96.35	93.87
	2	8,247,315,994	81,656,594	52.54	96.75	94.48

**Table 2:** RNA-Seq analysis statistics.

Sample	Replicate	Filtered reads	(%)	GC (%)	Aligned reads	(%)
Control	1	70,211,070	93.1	54.44	55,186,378	78.6
	2	75,292,976	95.7	52.37	60,098,440	79.8
Infection (Ra)	1	60,448,194	86.6	60.47	38,211,098	63.2
	2	73,020,706	94.4	52.51	61,890,146	84.8
Infection (Rv)	1	63,443,778	89.2	56.12	46,766,174	73.7
	2	77,642,052	95.1	52.39	66,843,776	86.1

**Figure 1:**
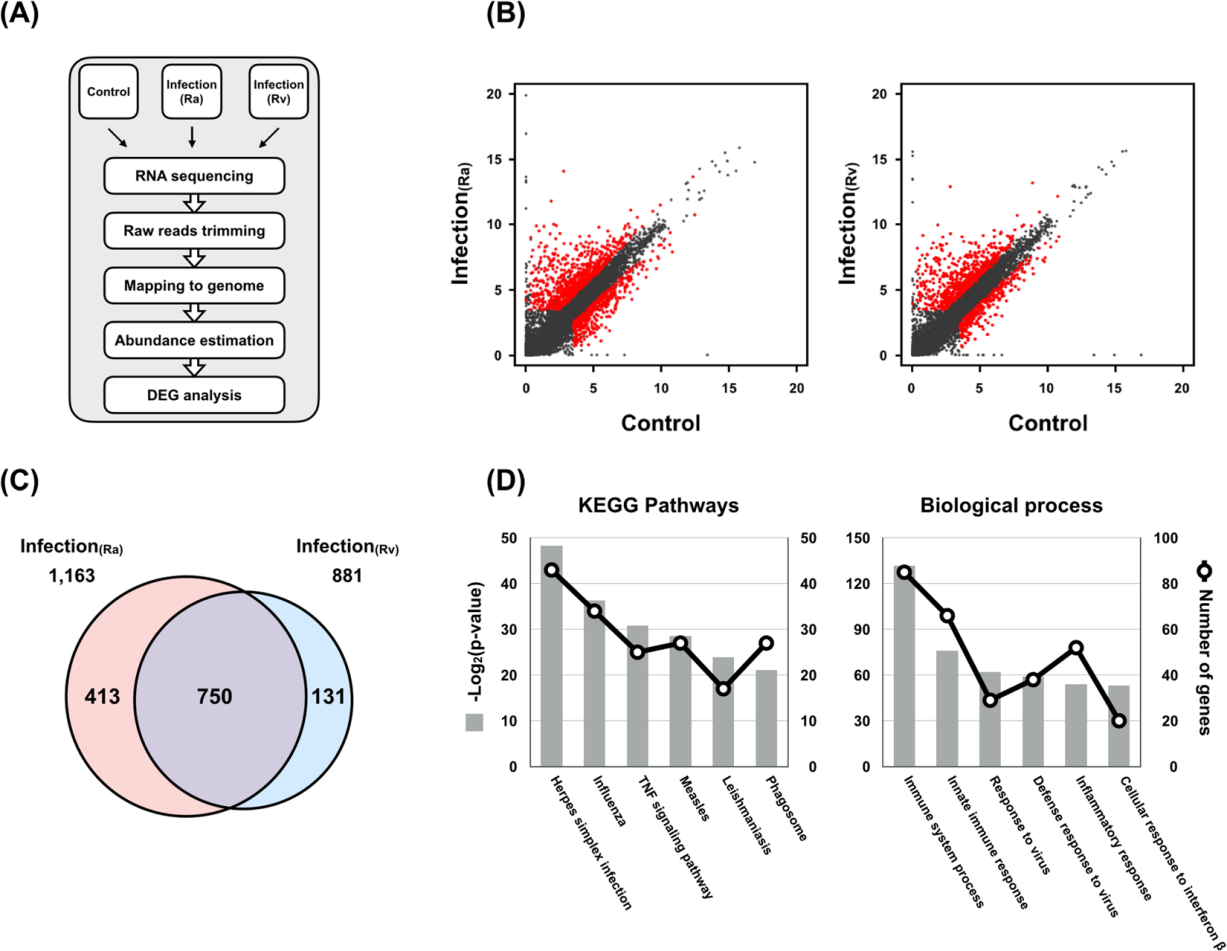
Transcriptome analyses of infected and non-infected samples. (**A**) Schematic diagram of the transcriptome analysis. (**B**) Scatterplot showing gene expression levels (log_2_-transformed fragments per kilobase of transcript per million mapped reads [FPKM]) between infection (Ra, H37Ra; Rv, H37Rv) and no infection samples (Control). Differentially expressed genes (DEGs) are marked in red. (**C**) Control. (**D**) The six most significantly enriched Kyoto Encyclopaedia of Genes and Genomes (KEGG) pathways (left) and Gene Ontology (GO) biological process categories (right) of the 750 genes differentially expressed in the control compared with Ra- or Rv-infected macrophages.

To identify virulence-associated transcriptional changes in Rv or Ra, we compared the transcriptome of macrophages infected with Rv or Ra with that of the control (Fig. 1A). In total, 1,163 or 881 genes were significantly differentially expressed (FPKM > 10 per sample and a false discovery rate (FDR) < 0.001) between Rv or Ra versus the control, respectively (Fig. 1B, Table S3). While 131 and 413 genes depended specifically on Rv and Ra, respectively, the remaining 750 genes were differentially expressed in both samples (Fig. 1C). Except for one gene, all the genes changed expression in the same direction in both Ra- and Rv-infected samples. Compared with the UN, 506 (67.6%) genes were upregulated in both Rv and Ra, whereas 243 (32.4%) genes were downregulated.

We next performed a functional classification and pathway analysis of 750 common differentially expressed genes (DEGs). Based on the Kyoto Encyclopaedia of Genes and Genomes (KEGG) pathway analysis, the six most enriched pathways were herpes simplex infection (mmu05168), influenza (mmu05164), tumour necrosis factor (TNF) signalling pathway (mmu04668), measles (mmu05162), leishmaniasis (mmu05140), and phagosome (mmu04145) (Fig. 1D). For example, TNF and its signalling pathway act to suppress bacterial growth, host cell death, and the production of pleural mesothelial cells (PMCs), which are the first-line defence against microorganism invasion^23,24^. Our data showed that 25 DEGs involving the TNF signalling pathway were significantly enriched 4.6-fold compared with the reference gene set (FDR = 6.59 × 10^−7^) (Table S2). Among them, Cxcl1, Cxcl2, Cxcl3, Cxcl10, IL1b, Socs3, and Mmp14 were overexpressed more than 100-fold compared with the non-infected sample regardless of infection type. These downstream genes of the TNF signalling pathway are key cytokines in leukocyte recruitment and the inflammatory response^25,26^ (see Supplementary Fig. S1). DAVID gene ontology analysis revealed that the TB pathway (mmu05152) was also significantly enriched (FDR = 0.0272) in our gene list (Table S2). The target genes were significantly enriched (FDR < 0.05) for biological process terms related to immune and inflammatory responses: immune system process (GO:0002376), innate immune response (GO:0045087), response to virus (GO:0009615), defence response to virus (GO:0051607), inflammatory response (GO:0006954), and cellular response to interferon-β (GO:0035458) (Fig. 1D). These results imply that 750 common DEGs may be involved in induction of the innate immune response and host defence mechanisms of the lung against mycobacterial infections.

To analyse how Mtb responds to the host environment, the remaining unaligned reads, which were not mapped to the mouse genome, were re-aligned to the Mtb reference genome. In total, 19,796 and 9,805 reads were mapped for the Ra and Rv samples, respectively. For the UN, only 216 reads were mapped to the Mtb reference genome, indicating that it was not a by-product of the alignment process. We found 124 genes (out of 3,976 genes in the Mtb genome) expressed in both Rv and Ra (Table S4). These genes were involved in phosphopantetheine binding (GO:0031177) and ATP binding (GO:0005524), which are likely associated with the proliferation of Mtb. Although 214 and 25 strain-specific genes (i.e. absent or significantly divergent in the Mtb genome strain) were identified, no GO categories were significantly (FDR < 5%) overrepresented (Table S4).

**Figure 2:**
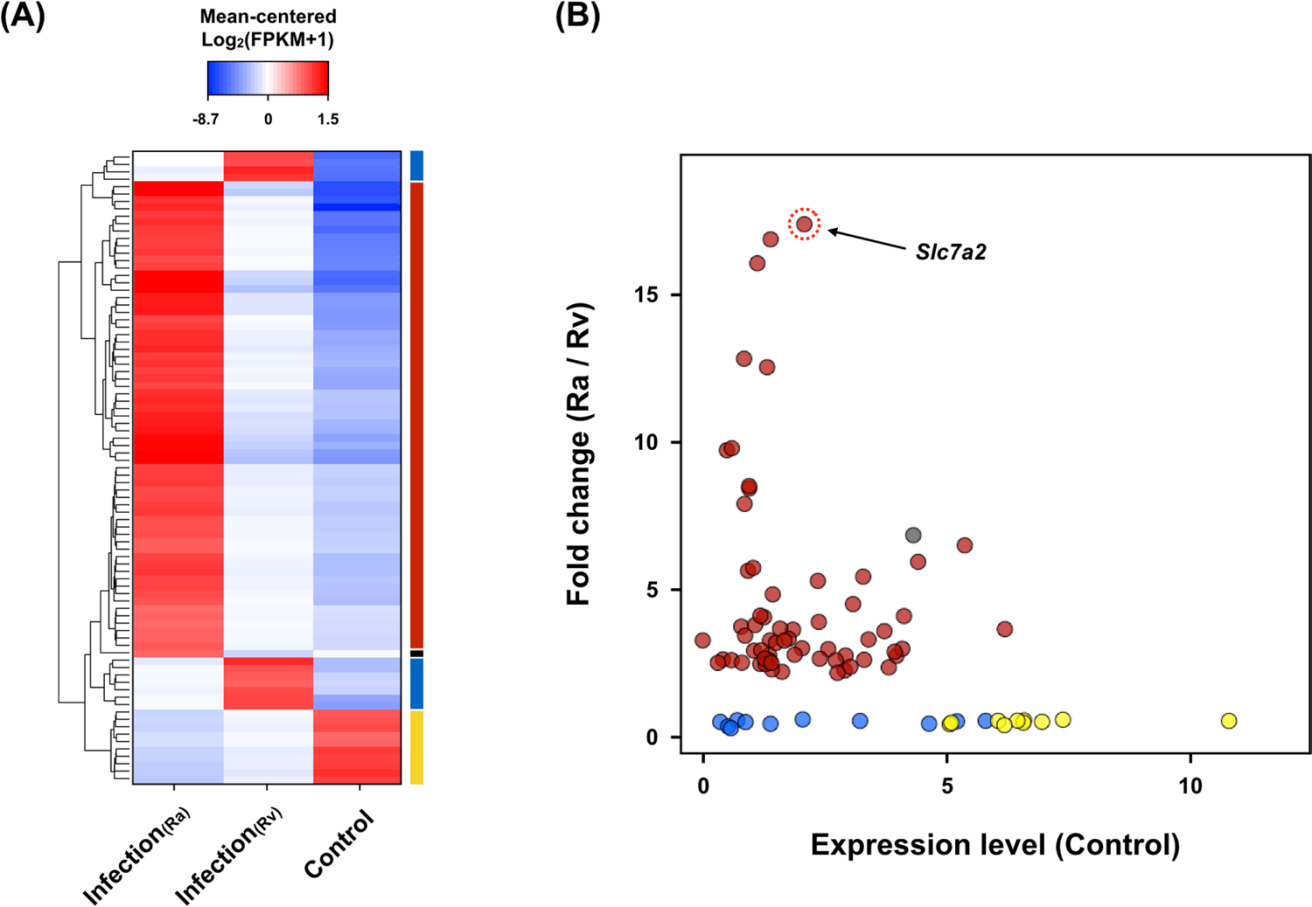
The DEGs between avirulent and virulent strains. (**A**) Heat map showing the expression patterns of 85 genes that were stimulated by *Mycobacterium tuberculosis* (Mtb) infection and differentially regulated between Ra- and Rv-infected samples. The colour bars on the right side indicate the four DEG groups: the expression levels are Control > Rv > Ra, yellow; Rv > Ra > Control, blue; Ra > Control > Rv, black; and Ra > Rv > Control, red. Hierarchical clustering of genes was performed with Euclidean distance matrices of normalised expression levels (mean-centred and log_2_-transformed FPKM). (**B**) *Slc7a2*, indicated with the red dotted circle, was the most overexpressed gene in the Ra-infected sample compared with the Rv-infected sample among 63 up-regulated genes. The dot colours are assigned as indicated in panel (A).

As there was strain-specific variation between Ra and Rv at the genomic and proteomic levels (PMID: 18584054, PMID: 27151218), we investigated how many of the transcriptional differences between Ra and Rv were attributable to discrimination. We examined the aforementioned 750 genes (Fig. 1C) and identified 85 that were differentially expressed between Ra and Rv infection. A majority of these genes (63/85, 74%) were expressed significantly more (FDR < 0.001) in Ra versus Rv (Fig. 2A). Among them, we found that *solute carrier family 7 member 2* (*Slc7a2*) was significantly overexpressed (about 17.4 times) in Ra compared with Rv (Fig. 2B). We postulate that this gene is related to the attenuation of Mtb and has functional roles via a host defence mechanism.

### SLC7A2 controls intracellular survival of mycobacteria in macrophages

**Figure 3:**
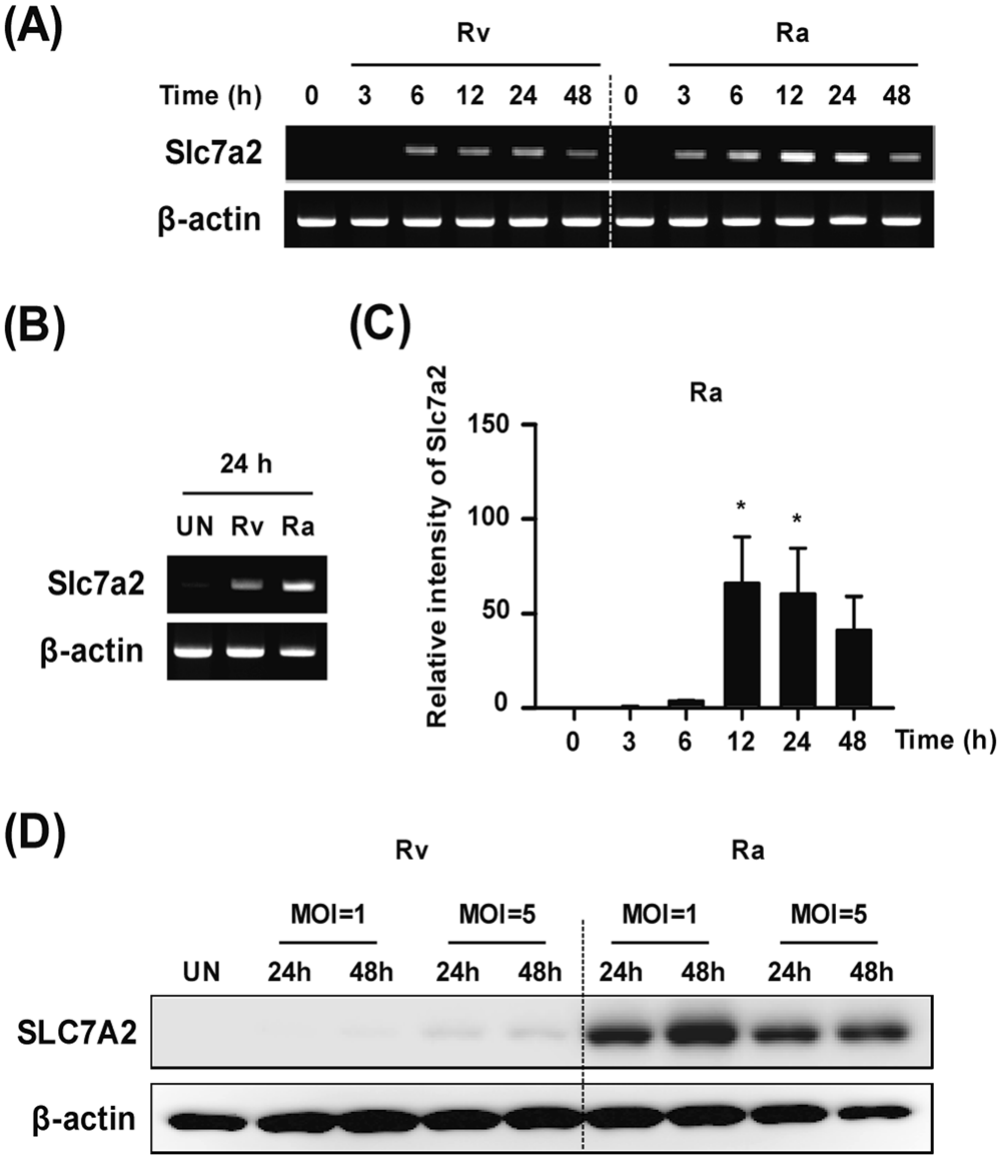
Differential induction of *Slc7a2* expression during mycobacterial infection. (**A**,**B**) Bone-marrow-derived macrophages (BMDMs) were infected with either Rv or Ra at a multiplicity of infection (MOI) of 1 for the indicated times. *Slc7a2* mRNA levels were analysed using reverse transcription PCR. (**C**) Ra infection-dependent *Slc7a2* expression was evaluated using quantitative real-time PCR in a time-dependent manner. (**D**) BMDMs were infected with Rv or Ra (MOI = 1 or 5) for the indicated times. After infection, SLC7A2 was detected using Western blotting. β-actin was used for the cell loading controls. Western blotting data are representative of three independent experiments. The data are the means ± SD of three independent experiments. *p < 0.05.

**Figure 4:**
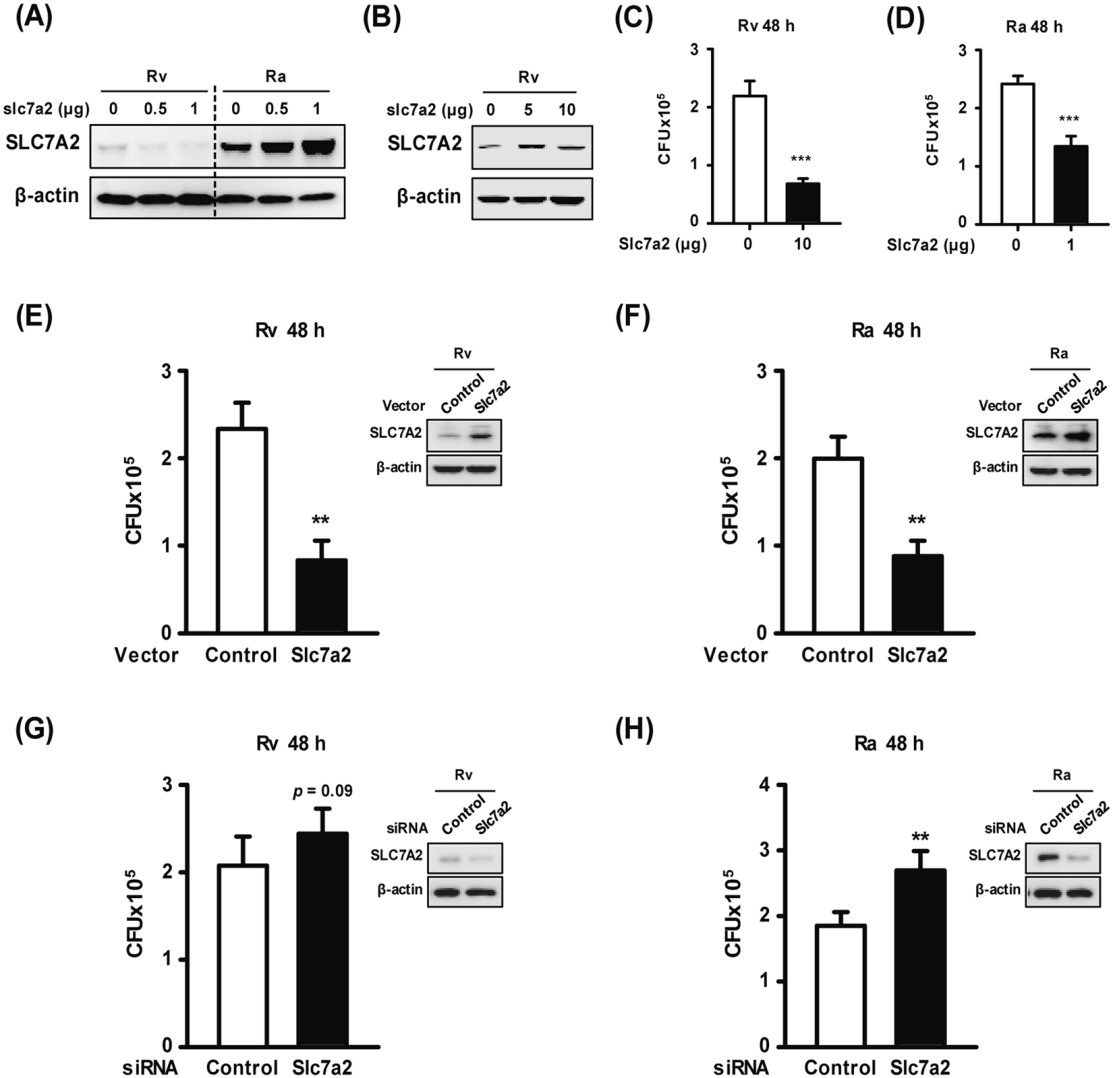
SLC7A2 expression controls intracellular mycobacteria survival. (**A**,**B**) BMDMs were transfected with pcDNA3.1-SLC7A2, infected with Mtb (MOI = 1) for 48 h, and analysed by Western blotting using anti-SLC7A2 antibody. (**C**,**D**) SLC7A2-overexpressed cells were infected with Rv or Ra (MOI = 1) for 48 h, after which intracellular bacterial survival was measured by CFU enumeration. (**E**) BMDMs were transfected with 10 μg of pcDNA3.1-SLC7A2 (Slc7a2) or pcDNA3.1 (Control), and were then infected with Rv (MOI = 1, 48 h). (**F**) For Ra infection (MOI = 1, 48 h), 1 μg of pcDNA3.1-SLC7A2 (Slc7a2) or pcDNA3.1 (Control) was used. (G and H) BMDMs were transfected with siControl (200 nM) or siRNA targeting *Slc7a2* (siSlc7a2 #3, 200 nM), and then infected with Rv or Ra (MOI = 1, 48 h). (**E**–**H**) Intracellular survival of mycobacteria was measured by CFU enumeration. The expression of SLC7A2 was detected by Western blotting. β-actin was used as a cell loading control. The Western blotting data presented are representative of three independent experiments. The data are the means ± SD of three independent experiments. **p < 0.01 and ***p < 0.001.

SLC7A2 is closely associated with inflammatory processes and infection^27^. Gene expression was significantly decreased in Rv-infected macrophages compared with Ra-infected macrophages. By contrast, it was highly up-regulated in Ra-infected macrophages compared with Rv-infected macrophages (Fig. 2B). To clarify the role of *Slc7a2* during mycobacterial infection, the temporal gene expression levels were examined by RT-PCR in Rv- and Ra-infected BMDMs. The expression level of *Slc7a2* was significantly increased later (12–24 h) after infection in Ra-infected macrophages compared with macrophages infected with Rv (Fig. 3A–C). Similarly, SLC7A2 protein levels were significantly lower in Rv-infected macrophages compared with those infected with Ra (Fig. 3D). To clarify the role of SLC7A2 during mycobacterial infection, the SLC7A2-overexpression vector was transfected into macrophages in a dose-dependent manner during Rv or Ra infection (Fig. 4A). The production of SLC7A2 was suppressed in Rv-infected macrophages despite the use of high-dose plasmid constructs (Fig. 4B). Next, the intracellular survival of Mtb in SLC7A2-overexpressing macrophages was measured 48 h after infection. The intracellular survival of Rv was significantly decreased in SLC7A2-overexpressing macrophages (0.6 ± 0.08 × 10^5^) compared with the control (2.1 ± 0.25 × 10^5^) (Fig. 4C). The intracellular survival of Ra in SLC7A2-overexpressing macrophages (1.3 ± 0.17 × 10^5^) was also significantly decreased compared with the control (2.4 ± 0.13 × 10^5^) (Fig. 4D). Similar to Fig. 4C,D, the intracellular survival of Mtb was reduced in SLC7A2-overexpressing macrophages comparing with the control vector-transfected macrophages (Fig. 4E,F).

To investigate the role of SLC7A2 in Mtb-infected macrophages, we silenced SLC7A2 expression using selected siRNAs targeting *Slc7a2* (see Supplementary Fig. S2A) and then infected the cells with Mtb. The phagocytosis of Rv or Ra did not differ between the SLC7A2 silencing and overexpressing conditions (see Supplementary Fig. S2B–E). As expected, intracellular survival of Rv was not significantly increased in siSlc7a2-transfected macrophages (2.4 ± 0.28 × 10^5^) compared with the control (2.0 ± 0.33 × 10^5^) (Fig. 4G), because SLC7A2 expression was suppressed in Rv-infected macrophages. By contrast, intracellular survival of Ra was significantly increased in siSlc7a2-transfected macrophages (2.6 ± 0.29 × 10^5^) compared with the control (1.8 ± 0.20 × 10^5^) (Fig. 4H). These results imply that Rv suppressed SLC7A2 expression to enable the virus to survive in macrophages.

## Discussion

In this study, we created genome-wide transcriptome profiles of host macrophages after infection with Rv or Ra using RNA-Seq. We produced 454 million RNA-Seq raw reads with an average of 2.7× genome coverage per sample. This coverage was sufficient to meet the minimum recommended coverage (1.8× ) for detecting all the expressed genes in the genome^28^, indicating that our high sequencing depth had a significant impact on measurements of overall expression levels, even of extremely low-abundance genes. Although the mapping rates between the replicas varied slightly, the number of uniquely mapped reads exceeded 100 million per sample, and our data show that gene expression levels were highly consistent (Tables 2 and S1).

Although many studies have focused on mycobacterial transcriptome analysis, little is known about the transcriptome of macrophages infected with virulent Rv and avirulent Ra. Our analysis compared infection-induced changes in gene expression in these two different Mtb strains. We found high expression of *Slc7a2* in Ra-infected macrophages compared with Rv-infected macrophages. SLC7A2 is a member of the cationic amino acid transporter (CAT) family, which transports arginine^29^. Inactivated macrophages require arginine transport for nitric oxide (NO) production via inducible nitric oxide synthase (iNOS)^30^. NO is important in the innate immune response against intracellular bacterial infection^31,32^. In CAT2 KO BMDMs, NO production was reduced compared with controls^33^. SLC7A2 expression induced NO generation in murine microglial cells^34^. NO effectively removed Mtb in murine macrophages^35–37^. Our results imply that *Slc7a2* is important for suppressing intracellular mycobacteria in macrophages because SLC7A2 is important for NO production, which leads to macrophage polarisation^38,39^. Of note, iNOS levels were enhanced in the granulomas of TB patients^40^. In peripheral blood mononuclear cells, NO production was increased in TB patients compared with normal controls^41^. iNOS expression has also been associated with an Mtb-killing effect in human alveolar macrophages and human monocytes^42,43^. Although some studies have shown that production of NO cannot remove Mtb in human macrophages^44,45^, NO might be effective against mycobacterial infection. In addition, *Leishmania* infection upregulated l-arginine transport via SLC7A2 in THP-1 cells^46^. Therefore, SLC7A2 plays an important role in some intracellular pathogen infections.

Several reports have shown that SLC7A2 KO mice exhibit suppressed expression of M1 phenotype markers such as TNFα, and iNOS^47–49^. M1 macrophages produce NO via the SLC7A2 transporter^38^. M1 macrophages effectively remove bacteria because they strongly induce the production of proinflammatory cytokines and NO^50–52^. Previously, we suggested that M1 is important to remove intracellular mycobacteria in macrophages^53^. Therefore, inhibition of Mtb by *Slc7a2* may be associated with M1 polarisation.

Summarising, we suggest that induction of *Slc7a2* is important in macrophages to regulate the intracellular survival of Mtb. Nevertheless, the exact function of *Slc7a2* as a host defence mechanism in TB remains to be clarified.

## Material and Methods

### Cells and bacterial culture

Wild-type mice (C57BL/6 background) aged 6–8 weeks were used in all experiments. Murine BMDMs were generated by flushing bone marrow cells from the femurs and tibias and culturing for 3–5 days in Dulbecco’s minimal essential medium (DMEM) containing 10% foetal bovine serum (FBS), penicillin (100 IU/mL), streptomycin (100 μg/mL), and 25 ng/ml macrophage colony-stimulating factor (M-CSF; R&D Systems, Minneapolis, MN, USA). All animal procedures were reviewed and approved by the institutional animal care and use committee of Chungnam National University, Daejeon, South Korea (permit no. CNU-00425). The animal experiments were performed in accordance with the Korean Food and Drug Administration guidelines.

Mtb Rv strain (ATCC27294) and Ra strain (ATCC 25177) were grown in Middlebrook 7H9 liquid medium containing 10% OADC (oleic acid, albumin, dextrose, and catalase) and 5% glycerol. The bacterial cells were suspended in phosphate-buffered saline (PBS) at a concentration of 1 × 10^8^ CFU/ml, and stored at −80 °C.

### Mtb infection and intracellular bacterial counts

Cells were infected with live Mtb at a multiplicity of infection (MOI) of 1 or 5 and incubated for 3 h at 37 °C, 5% CO_2_. After allowing time for phagocytosis, the cells were washed with PBS to remove the extracellular bacteria before incubation with fresh medium without any antibiotics.

To measure the survival of intracellular Mtb, BMDMs were infected with Mtb for 48 h and lysed in distilled water to collect the intracellular bacteria. The lysates were serially diluted in 7H9 broth, plated separately on 7H10 agar plates, and incubated for 2–3 weeks at 37 °C. Colony counting was then performed in triplicate.

### PCR and Western blot analysis

Total RNA was isolated from Mtb-infected BMDMs and mRNA was reverse transcribed into cDNA. Reverse transcription PCR was performed using Prime Taq Premix (Genet Bio, Daejeon, South Korea) to detect the mRNA levels of target genes. For quantitative real-time PCR, cDNA was synthesised and target gene expression was quantified by SYBR green (QIAGEN, Venlo, The Netherlands). The mean value of triplicate reactions was normalised against the mean value of β-actin.

For Western blotting, Mtb-infected cells were lysed in radioimmunoprecipitation (RIPA) assay buffer containing protease inhibitor cocktail (PIC), and whole-cell lysates were separated on a 12% SDS-PAGE gel before being transferred to a polyvinylidene difluoride (PVDF) membrane. Primary antibodies against SLC7A2 (Abcam, Cambridge, UK) were diluted 1:1,000 overnight at 4 °C, and goat anti-rabbit IgG (Santa Cruz, Dallas, TX, USA) was used as a horseradish peroxidase (HRP)-conjugated secondary antibody and was diluted 1:2,000 for 2 h at room temperature. β-actin (1:5,000, Santa Cruz) was used as a loading control. To detect the target proteins, membranes were developed using a chemiluminescent HRP substrate reagent (ECL, Millipore, Billerica, MA, USA) and subsequently quantified using the Alliance Mini 4 M (UVitec, Cambridge, UK).

### Overexpression and RNA interference

SLC7A2 was overexpressed using vectors synthesised by Cosmogenetech (Seoul, South Korea). Constructed plasmids were confirmed by sequencing by Cosmogenetech and were used in the experiments. BMDMs were transiently transfected with the mouse SLC7A2 (pcDNA3.1-SLC7A2) expression vector or with an empty vector (pcDNA3.1) using Lipofectamine 3000 (Invitrogen, Carlsbad, CA, USA). siRNA targeting *Slc7a2* (Bioneer Corporation, Daejeon, South Korea) was used to silence the expression of proteins. The BMDMs were transiently transfected with mouse siSlc7a2 or siControl using Lipofectamine 3000 (Life Technologies, Grand Island, NY, USA), according to the manufacturer’s protocol. After overnight incubation, vector- or siRNA-transfected cells were infected with Mtb before the expression levels of the target protein and the intracellular survival of the bacteria were measured.

### Preparation of the total RNA library and sequencing

Total RNA from Mtb-infected BMDMs was extracted with TRIzol reagent according to the manufacturer’s instructions. The RNA was quantified and assessed for purity by a 2100 Bioanalyzer (Agilent Technologies, Waldbronn, Germany). A sequencing library was prepared with 1 μg of total RNA for each sample using the Illumina TruSeq™ RNA Sample Preparation Kit (Illumina, San Diego, CA, USA). In brief, the total RNA sample was treated with the Ribo-Zero rRNA Removal Kit (Epicentre, Madison, WI, USA) to deplete bacterial and eukaryotic ribosomal RNA (rRNA). The remaining RNA was converted to single-stranded cDNA using reverse transcriptase and random hexamers and the second strand was synthesised to produce double-stranded cDNA ready for application to TruSeq library construction using DNA Polymerase I and RNase H. These cDNA fragments underwent an end repair process, the addition of a single ‘A’ base, and ligation of the indexing adapters. The products were purified and enriched with PCR to create the final cDNA library. Finally, the libraries were quantified and qualified using a qPCR quantification protocol guide (KAPA Library Quantification Kits for Illumina Sequencing platforms) and TapeStation D1000 ScreenTape (Agilent Technologies), respectively. The resulting cDNA libraries were sequenced using the HiSeq2500 platform (Illumina), generating approximately 453 million paired-end reads of 101 nucleotides in length. All raw sequence data have been deposited in the NCBI database with the accession numbers SRX5057205-SRX5057210 under BioProject PRJNA50644.

### Transcriptome and bioinformatics analysis

Figure 1A presents the experimental procedures for the transcriptome analysis. All raw sequence reads by RNA-Seq from each tissue sample were initially pre-processed by Trimmomatic (v0.36)^54^ to trim the adaptor sequences and remove low-quality sequences. The remaining clean reads were mapped to the mouse and Mtb genomes using Tophat2^55^. To quantify the known transcripts in mouse and two *M*. *tuberculosis* strains (virulent Rv and attenuated Ra), the alignment results were input into Cufflinks (v2.2.1)^56^. Unless otherwise stated, the unit of expression level in our analyses is FPKM. Cuffdiff (v2.2.1)^57^ was used to test for differential expression. We defined genes as differentially expressed using the following criteria: FPKM > 10 and FDR < 0.001. The data for visualisation were generated by R (R Development Core Team, Vienna, Austria). The genome sequences and the annotations of mouse (mm10) and two *M*. *tuberculosis* strains were obtained from the UCSC genome browser (https://genome.ucsc.edu) and the *Mycobacterium tuberculosis* Genome Database (https://mycobacterium.biocyc.org), respectively. We analysed differences in the enrichment of Gene Ontology (GO) categories and KEGG pathways for the DEGs using the DAVID (v6.8) functional annotation analysis tool (https://david.ncifcrf.gov)^58^.

## Supplementary information

Supplementary Figures

Supplementary Table S1

Supplementary Table S2

Supplementary Table S3

Supplementary Table S4
