# Supplementary material for: Characterisation of genes differentially expressed in macrophages by virulent and attenuated Mycobacterium tuberculosisthrough RNA-Seq analysis
Source: Sci Rep. 2019 Mar 11;9:4027. doi: 10.1038/s41598-019-40814-0 (PMC6411972; doi:10.1038/s41598-019-40814-0)
Supplement: Supplementary file 1 — Supplementary Figures [file 41598_2019_40814_MOESM1_ESM.pdf]

Characterization of genes differentially expressed within macrophages by virulent and attenuated *Mycobacterium tuberculosis* through RNA-Seq analysis

Running title: SLC7A2 suppressed intracellular survival of Mtb

Junghwan Lee[1,2,#], Sung-Gwon Lee[5,#], Kee K. Kim[4], Yun-Ji Lim[1,2,3], Ji-Ae Choi[1,2,3], Soo-Na Cho[1,2], Chungoo Park[5*] and Chang-Hwa Song[1,2,3*]

[1]Department of Microbiology and [2] Department of Medical Science, [3] Research Institute for Medical Sciences, College of Medicine, Chungnam National University, Daejeon 35015, South Korea

[4]Department of Biochemistry, Chungnam National University, Daejeon, 34134, South Korea.

[5]School of Biological Sciences and Technology, Chonnam National University, Gwangju 61186, South Korea

[#]These authors contributed equally to this work.

[*]Correspondence: Chungoo Park, Ph.D., School of Biological Sciences and Technology, Chonnam National University, Gwangju 61186, South Korea. E-Mail: chungoo@jnu.ac.kr and Chang-Hwa Song, Ph.D., Department of Microbiology, College of Medicine, Chungnam National University, Daejeon 35015, South Korea.

E-Mail: songch@cnu.ac.kr

Supplementary information

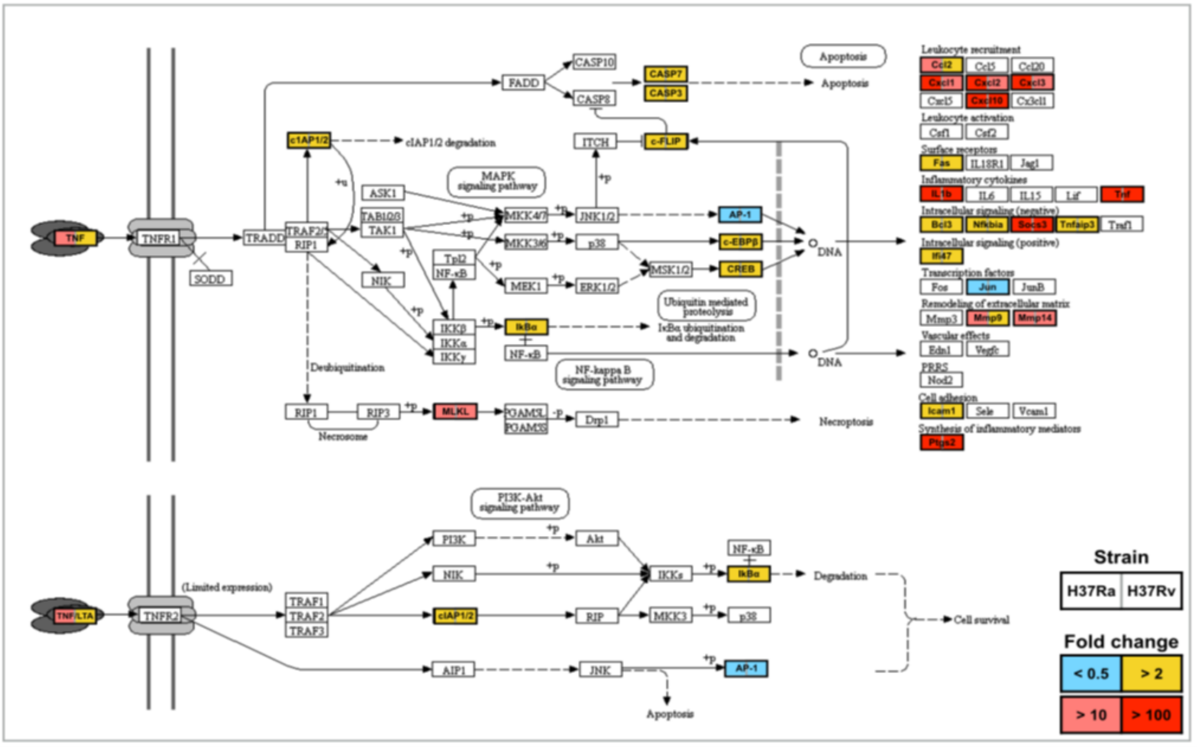

Figure S1. 25 differentially expressed genes involved in TNF signaling pathway in the KEGG database. The left and right sides of the box represented gene expression fold-changes between control versus the H37Ra and H37Rv strains, respectively. For an extensive list of genes induced by TNF, we refer to the Kyoto Encyclopedia of Genes and Genomes (KEGG), pathway map 04668. URL:http://www.genome.jp/. The KEGG database has been described previsouly.[58]

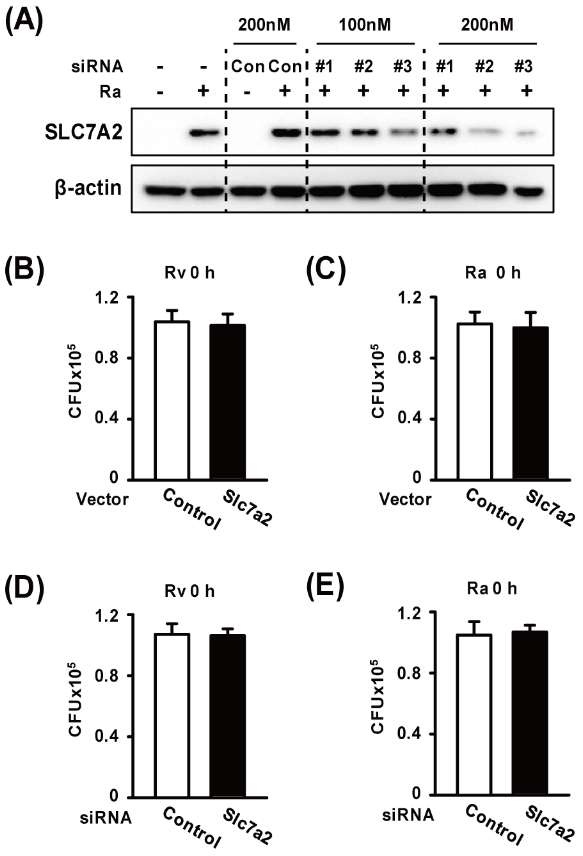

Figure S2. Mtb-induced SLC7A2 expression in macrophages.

(A) BMDMs were transfected with one of three different siRNAs targeting *Slc7a2* (#1, #2 and #3) or siControl (Con) and then were infected with Ra (MOI=1, 48 h). After infection, SLC7A2 was detected by Western blotting. β-actin was used for the cell loading controls. (B) BMDMs were transfected with 10 μg of pcDNA3.1-*Slc7a2* (Slc7a2) or pcDNA3.1 (Control) and then were infected with Rv (MOI=1). (C) BMDMs were transfected with 1 μg of Slc7a2 or control and then were infected with Ra (MOI=1). (D and E) BMDMs were transfected with 200nM of siRNA (siControl or siSlc7a2 #3) and then were infected with Rv or Ra (MOI=1). (B-E) After infection 3 h, phagocytosis of Rv or Ra was measured by CFU assay.

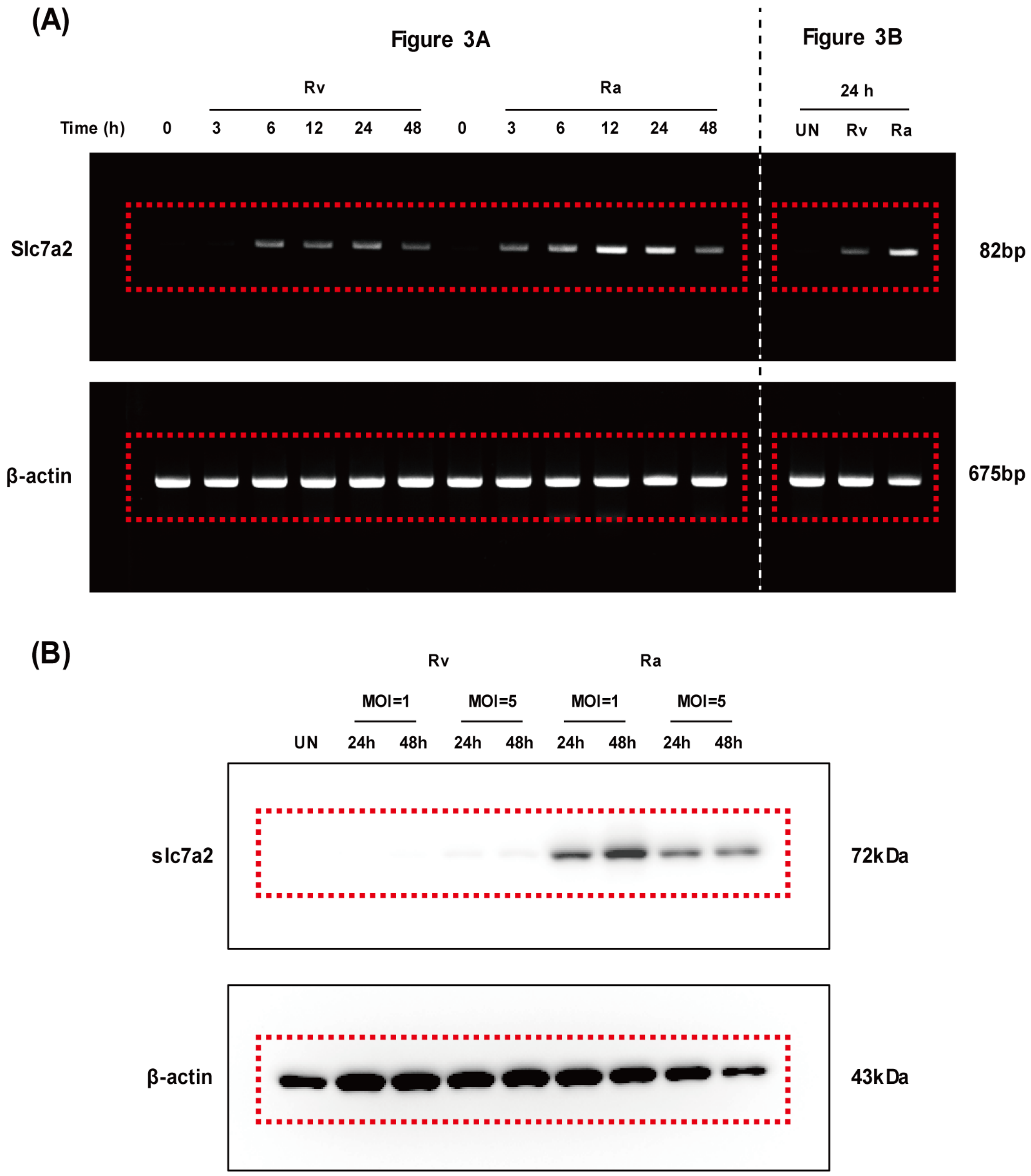

Figure S3. Full length gel and blot of figure 3.

(A) Full length gel of (left) figure 3A and (right) 3B. (B) Full length blot of figure 3D. Red dotted lines show the cropping.

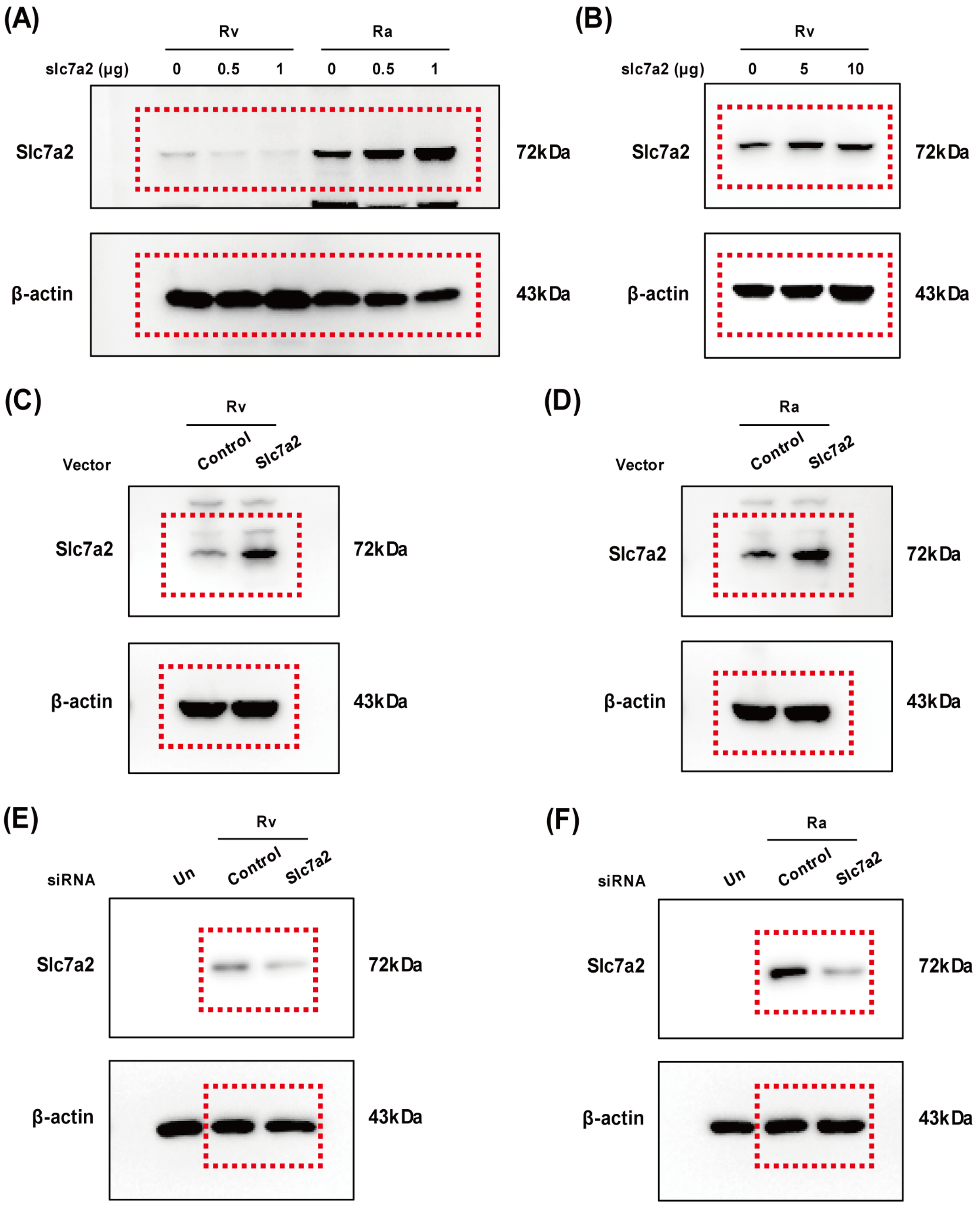

Figure S4. Full length blot of figure 4.

Original membranes for the representative Western Blots shown in (A) figure 4A, (B) figure 4B, (C) figure 4E, (D) figure 4F, (E) figure 4G and (F) figure 4H. Red dotted lines show the cropping.

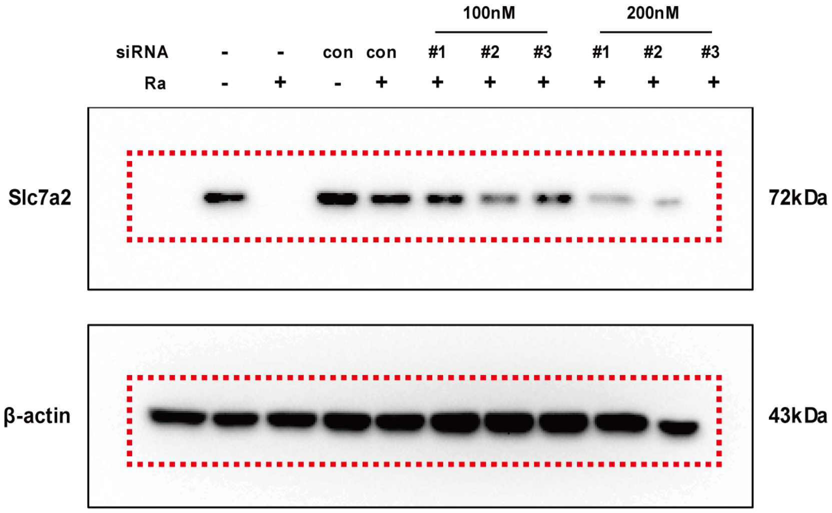

Figure S5. Full length blot of Supple figure 2A.

Red dotted lines show the cropping.
